# Total Pesticide Exposure Calculation among Vegetable Farmers in Benguet, Philippines

**DOI:** 10.1155/2009/412054

**Published:** 2009-11-05

**Authors:** Jinky Leilanie Lu

**Affiliations:** National Institutes of Health, University of the Philippines Manila, Ermita, Manila 1100, Philippines

## Abstract

This was a cross-sectional study that investigated pesticide exposure and its risk factors targeting vegetable farmers selected through cluster sampling. The sampling size calculated with *P* = .05 was 211 vegetable farmers and 37 farms. The mean usage of pesticide was 21.35 liters. Risk factors included damaged backpack sprayer (34.7%), spills on hands (31.8%), and spraying against the wind (58%). The top 3 pesticides used were pyrethroid (46.4%), organophosphates (24.2%), and carbamates (21.3%). Those who were exposed to fungicides and insecticides also had higher total pesticide exposure. Furthermore, a farmer who was a pesticide applicator, mixer, loader, and who had not been given instructions through training was at risk of having higher pesticide exposure. The most prevalent symptoms were headache (64.1%), muscle pain (61.1%), cough (45.5%), weakness (42.4%), eye pain (39.9%), chest pain (37.4%), and eye redness (33.8%). The data can be used for the formulation of an integrated program on safety and health in the vegetable industry.

## 1. Introduction

Agriculture has been one of the primary economic avenues in the Philippines contributing to about 20% to the gross domestic product (GDP). Crops comprise about 47.56% of the total agricultural sector and have contributed to about 510 billion pesos (P510B) to the country's national income [1].

Benguet is a province in the northern portion of the Philippines belonging to the Cordillera Administrative Region. It has about 2599.4 km^2^ of land area with a population of 372 533. Agriculture makes up the province's main economic revenue. There are about 27.5 thousand farms covering 30 thousand hectares of agricultural land in Benguet. It is also the largest producer of vegetables and fruits, supplying the capital cities in the Philippines. The province is known as the “salad bowl” of the Philippines as its major crops are tubers, roots, bulbs, leafy vegetables, stems, and flowers. In 2005, Benguet was the top producer of Brocolli and carrots producing about 1.2 thousand and 13.7 metric tons contributing to 87.4% and 81.4%, respectively, to the national output [1]. However, growing vegetables is considered a risk occupation in some areas in developing countries. Soogarun et al. in 2003 [2] found significantly low/abnormal mean blood cholinesterase levels among vegetable growers in Thailand.

Health impacts of pesticide misuse on the other hand greatly affect the farming communities in the Philippines questioning the economic advantages of its use. Many researchers have correlated the extent of direct and indirect pesticide exposure and health hazards such as increased mortality, dermal contamination, depression in cholinesterase level, fetal abnormalities, and spontaneous abortion among pregnant women [3–6]. It is a discouraging fact though, that with knowledge of health risks, many Filipino families still perceive that crop yield outweighs the health risks associated with pesticide use.

Pesticide poisoning is one of the most prevalent health problems in the Philippines. In a study by the Department of Health (DOH) from 1991–1995, organophosphates accounted for the highest number of poisoning cases while organochlorines caused the most number of deaths [7]. Other Philippine studies related to pesticide poisoning show the adverse health effects of pesticide. Cheng in 1994 [8] studied 2000 Benguet vegetable farmers and found that the most common complaints were allergic reactions both in the skin and the eyes, abdominal pain, dizziness, chest pain, headache, and nose bleed. Meanwhile, a study on pesticide poisoning in selected hospitals in four Philippines regions in 2001 found that cases of acute poisoning were more prevalent than chronic cases [8].

This study aimed to identify the pesticide exposure and risk factors among vegetable farmers. The data can be used as baseline data on the vegetable industry in the Philippines.

## 2. Methodology

This was a cross-sectional study to investigate the prevalence of pesticide exposure and its risk factors. Target population consisted of vegetable farmers in the largest vegetable producing community in the Philippines. The inclusion criteria were farmers living in the community for at least one year from the time of interview, and practicing farmers who own or work a farm in the community. Those who were involved in organic farming and the migrant farmers who have been in the area for less than one year were excluded. There were 211 respondents from the identified municipalities selected as the study population using cluster sampling. The sampling size calculated with *P* = .05 was 211 vegetable farmers and 37 farms.

Data gathering was done using the following: (1) questionnaire—structured personal interview with farm workers/farmers was done by research assistants who were trained prior to the data collection. Details included personal information, health history, pesticide usage, work practices, work conditions, risk factors associated with pesticide exposure, and health data; (2) exposure assessment monitoring on work conditions, work practices, and pesticide concentration; (3) work analysis in each farm was also done to validate work practices related to pesticide preparation and application. Recall bias was dealt with by confining the health data questionnaire to the last one year from the time of interview. The health data were also collected by medical doctors who simultaneously conducted physical assessment of the farmers.

Data were encoded and analyzed using SPSS program. Statistical tools used were summary statistics, and Pearson correlation coefficient.

This project study was collaborated with the local agencies coordinating with farmers in the vegetable industry in Benguet. Ethical clearance was given by the Research, Information and Dissemination Office of the proponent's Institute.

## 3. Results and Discussion

### 3.1. Sociodemographic Profile

The study included 127 males (60.2%) and 84 females (39.8%) with ages ranging from 16 to 72 (mean = **45** ± **12**) showing a relatively adult population. Seventy one percent (71%) were married and majority were working as agricultural workers (82%), and the remaining were pesticide applicators, mixers, and loaders (18%). The respondents were living in their present address for an average of 34.76 years (SD = ±**16.72**) with a mean distance of 3163 meters (SD = ±**36539.13**) from the vegetable plantation or farm.

Few farmers reported history of smoking (16.2%), and 7% claimed they smoked and 2% had a history of chewing tobacco. The average number of cigarettes and tobacco consumed in a week were 12 sticks and <1 tobacco, respectively.

### 3.2. Factors Related to Pesticide Exposure

The farmers used pesticides in their farms in an average of 1.9 days per week. The mean total application time was 3.47 hours (mean = 3.47 ± 2.09). The mean amount of pesticide used in an application was 21.35 L per application (mean 21.35 ± 48.17). The farmers also reported that in an average year, there were 2.3 (mean = 2.3 ± 0.53) cropping seasons with a mean of 3.84 (mean = 2.3 ± 0.53) months per cropping season (Table 1).

72% had spills while they were spraying, mixing and loading. 44.5% reported that they wiped their sweat with a contaminated piece of fabric, 41.7% re-entered recently sprayed area, 37.4% had exposure because of damaged backpack sprayer, and 31.8% were exposed when they sprayed against the wind (Table 2).

 One hundred seventy six or 88.4% reported that they wore protective equipment while working. However, further analysis shows that they did not frequently use such equipment nor had adequate gear to fully protect themselves. One hundred forty two or 67% never used coveralls. The same pattern was seen among all kinds of personal protective equipment (PPE) with the exception of boots which was frequently used by 77.5% of farmers.

### 3.3. Pesticide Exposure

94% said that they have worked with or used pesticides in their lifetime, and 16.4% from this population used pesticides in their own households. The vegetables commonly grown in the area were potatoes (67.4%), cabbage (63.7%), and carrots (36.8%).

Majority (87%) reported occupational exposure to pesticides during their farm work while 13% were exposed accidentally. The predominant form of exposure was liquid mist (56.5%). The most common route of pesticide entry in the study was respiratory (68.9%) followed by dermal and ocular entry (60.5% and 38%, resp.).

Majority of the respondents used pyrethroid (46.4%) in their agricultural work. 24.2% said they used organophosphates while 21.3% used carbamates.

Sumicidine was the most commonly used pyrethroid which contains fenvalerate as its active ingredient. Meanwhile, Methamidophos is an active ingredient in most organophosphate pesticides. Mancozeb is present in carbamates while cypermethrin is found in pyrethroids.

Table 3 shows that 37% and almost 14.7% of the study population used pesticides with active ingredients of fenvalerate and cypermethrin, respectively. Both of these ingredients are classified by WHO as moderately dangerous. 24.2% of the farmers used methamidophos which is a highly dangerous formulation. 23.7% used mancozeb which is not dangerous under normal use.

Although pyrethroid was the most frequently used pesticide, it is organophosphate that consisted the largest amount of exposure among farmers at 210.02 liters, followed by pyrethorid at 151.4 liters, and carbamates at 32.16 liters for the entire one year.

The pesticide exposure of the farmers measured in Table 4 as dependent variable was related to the amount of pesticide used in liters, frequency of use and duration of use. All the independent variables, except amount and years of pesticide use were categorical variables. Those who used more pesticides over a longer period of time had higher total pesticide exposure. Those who were exposed to fungicides and insecticides also had higher total pesticide exposure. Furthermore, a farmer who was a pesticide applicator, mixer, loader and who wiped sweat with contaminated piece of fabric, and who had not been given instructions through training association was at risk of having higher pesticide exposure.

Seventy four percent (74%) of the respondents became ill because of work for the last 12 months preceding the study. The most common symptoms were headache (64.1%), muscle pain (61.1%), cough (45.5%), weakness (42.4%), eye pain (39.9%), chest pain (37.4%), and eye redness (33.8%). These health symptoms were non-specific for pesticide exposures. A subsequent study is recommended to focus on adverse health effects of these farmers and association with certain risk factors.

## 4. Discussion

The results of this study identified pesticide exposure and farming practices of farmers in the largest vegetable producing area in the Philippines. The poor PPE use was seen in this study. This has also been documented in other countries. In the study of Coble et al. in 2005 [9], and Thompson et al. in 2003 [10], poor usage of protective equipment increases pesticide residues accumulating in the body.

Unsafe practices like re-entry of recently spayed area, use of damaged backpack sprayer and wiping sweat with a contaminated piece of fabric were identified in this study. Re-entering a recently sprayed area, as mentioned in the study of Tielemans et al. in 1999 [11] is an important determinant of dermal exposure to specific chemicals such as captan and tolylfluanid.

A pesticide's formulation is a significant factor for human exposure, with greater risks present among aqueous and emulsifiable concentrates because it impairs the protective function of chemically protective gloves [12]. According to Wolfe in 1993 [13], pesticides may react through chemical and biotic processes. However, pesticides may undergo activation processes unexpectedly and may be broken down to equally or more potent and mobile toxic compounds posing a greater threat to nontarget organisms. It is then advised to limit or decrease the frequency or duration of staying in the contaminated crops right after pesticide application.

Organophosphates, carbamates and pyrethroid pesticides were the most commonly used type of pesticides among the farmers in this study. The same was seen in the study of Clarke et al. in 1997 [14] in Ghanna where organophosphates consisted of the most commonly used pesticides followed by carbamates and organochlorines. The same trend was seen among farmers in Sri Lanka and in Brazil [15, 16].

The farmers in this study used pesticides in their farms with a mean application time of 3.47 hours (mean = 3.47 ± 2.09). The mean amount of pesticide used in an application was 21.35 liters per application (mean 21.35 ± 48.17). The number of spray operations per week has been proven to have significant association with the likelihood of experiencing neurobehavioral, respiratory, or intestinal symptoms in a study among Indonesian farmers [17]. In a study among North Carolina growers and agents [18], it was found that the study population perceived that once the pesticide is diluted and reentry intervals are observed, the risk it poses becomes diminished.

Sumicidine, which was the most commonly used pesticides among the Benguet farmers contains fenvalerate. Fenvalerate induces numbness, itching, tingling, and burning sensations in exposed workers that developed after a latent period of approximately 30 minutes, peaked by 8 hours, and disappeared within 24 hours [19]. Additional data among Chinese workers demonstrated that fenvalerate decreased the semen quality of occupational workers [20].

On the other hand, other active ingredients like cypermethrin, mancozeb, and methamidophos have documented effects to humans. Skin sensations were reported to occur among field workers and usually lasted only for a few hours and did not persist for more than one day after exposure to cypermethrin [19]. For mancozeb, prolonged low-level exposure to mancozeb affected several aspects of immune functioning [21] and moderate association existed between mancozeb and neural tube defects [22].

Farmers also used inappropriate clothing or equipment for protection. Most often, gloves were the most commonly used personal protective equipment because the hands were the most exposed areas [23, 24]. Many circumstances contributed to nonadherence to proper use of PPE like extreme heat during pesticide application, uncomfortable to use, few resources to afford new PPE, peer-related factors, and increasing age [18, 25–27].

The study also showed certain risk factors associated with pesticide exposure such as re-entering recently sprayed area, spraying against the wind, use of damaged backpack sprayer, spills on the back, spills while mixing pesticides, among others. Aside from direct pesticide use, the different agricultural tasks mentioned above may also contribute as risk factors to pesticide exposure. Re-entering a recently sprayed area, as mentioned in the study of Tielemans et al. in 1999 [11] is an important determinant of dermal exposure to specific chemicals such as captan and tolylfluanid.

There are many health symptoms associated with pesticide exposure. There is evidence that weight loss could be a possible health effect of chronic pesticide poisoning. Decreased body mean mass accompanied by reduced cholinesterase activities among seven farm workers was documented [28]. Also, Kackar et al. in 1999 [29] found that when rats were administered orally with mancozeb (ethylenebisdithiocarbamate), dose-dependent signs of poisoning, weight loss, and mortality developed.

Respiratory symptom such as coughing were also documented in this study. Senthilselvan et al. found a significant association between carbamate exposure and prevalence of asthma among those non-asthmatic farmers and lower mean lung function variables among those with asthma [30].

This study has shown the pesticide exposure of farmers in the largest vegetable producing area in the Philippines. It is vital that a sequential exposure assessment be done in order to come up with a correlation study between pesticide exposure and health problems.

## 5. Conclusion

The study showed that pesticide use is prevalent among farmers in Benguet which is the largest vegetable producer in the Philippines. There were unsafe work practices that predisposed the farmers to health related problems. This study suggests that intervention measures be done to lower pesticide exposure of farmers. It is also suggested that chronic effects of pesticide cited in certain studies [31, 32] such as carcinogenic effects, poor reproductive outcomes, neurologic and respiratory disorders, impairments of the immune system and birth defects should also be investigated in future studies.

This manuscript adds to existing literature on pesticide exposure in the Philippines which are so far mainly descriptive in nature. This paper also identifies risk factors such as work practices and designs of containers/sprayers that may increase pesticide exposure among farmers. This also calls for a local level policy research for program intervention among vegetable farmers using pesticides.

## Figures and Tables

**Table 1:** Mean pesticide exposure among vegetable farmers in Benguet.

Pesticide usage in farm	Mean	Standard deviation
Amount of pesticide (Liters) per application	21.35	48.17
Average of total application time (hrours) per day	3.47	1.84
Average amount of time used to prepare dilution (minutes) per application	14.18	29.88
Average spraying application per day	2.76	3.60
Days of pesticide use in a week	1.90	1.23
Months per cropping season	3.84	0.75
Cropping seasons per year	2.30	0.53

**Table 2:** Percentage distribution of work practices.

Risk factors	Frequency	Percentage (%)
Given instructions on how to use pesticide	156	73.9
Spills while spraying	152	72.0
Spills while mixing and loading	152	72.0
Wiping sweat on the face with a contaminated piece of fabric	94	44.5
Re-enter recently sprayed area	88	41.7
Damaged backpack sprayer	79	37.4
Spraying Against the wind	67	31.8
Eating at Worksite	11	5.2

**Table 3:** Frequency Distribution of Active Ingredients of Pesticides Used. IA: extremely dangerous; IB: highly dangerous; II: moderately dangerous; III: slightly dangerous; 0: not dangerous under normal use.

Pesticide classification	Active ingredient	WHO classification	Frequency	Percentage
Pyrethroid	Fenvalerate	II	78	37.0
	Cypermethrin	II	31	14.7
Organophosphate	Metamidophos	IB	51	24.2
Carbamates	Mancozeb	O	50	23.7

**Table 4:** Bivariate Analysis between Total Pesticide Exposure and Certain Risk Factors. Correlation significant at *P* = .05 level.

Exposure factors	Pearson correlation coefficient (*P* value)
Amount of pesticide used (in liters)	.465 (.001)
Years of pesticide use	.247 (.003)
Wiping sweat with contaminated piece of fabric	.155 (.049)
Insecticides	.351 (.001)
Fungicides	.177 (.025)
Instructions given through trainings	.167 (.036)
Agricultural pesticide applicator/mixer/loader	.180 (.023)

## References

[1] Bureau of Agricultural Statistics 2008 Crop Statistics. http://www.bas.gov.ph/.

[2] Soogarun S, Wiwanitkit V, Suwansaksri J (2003). Report on blood cholinesterase among vegetable growers. *Southeast Asian Journal of Tropical Medicine and Public Health*.

[3] Loevinsohn ME (1987). Insecticide use and increased mortality in rural Central Luzon, Philippines. *The Lancet*.

[4] Ambridge EM, Haines IH, Lambert MRK (1990). Operator contamination during pesticide application to tropical crops. *Medicina del Lavoro*.

[5] Ostrea EM, Bielawski DM, Posecion NC (2008). A comparison of infant hair, cord blood and meconium analysis to detect fetal exposure to environmental pesticides. *Environmental Research*.

[6] Crisostomo L, Molina VV (2002). Pregnancy outcomes among farming households of Nueva Ecija with conventional pesticide use versus integrated pest management. *International Journal of Occupational and Environmental Health*.

[7] Department of Health Deaths and CFR of various pesticide poisoning in four regions in the Philippines.

[8] Cheng C (1994). Pesticides and its hazardous effects on the Benguet vegetable farmers. *Journal of Pesticide Management*.

[9] Coble J, Arbuckle T, Lee W, Alavanja M, Dosemeci M (2005). The validation of a pesticide exposure algorithm using biological monitoring results. *Journal of Occupational and Environmental Hygiene*.

[10] Thompson B, Coronado GD, Grossman JE, Puschel K (2003). Pesticide take-home pathway among children of agricultural workers: study design, methods, and baseline findings. *Journal of Occupational and Environmental Medicine*.

[11] Tielemans E, Louwerse E, de Cock J (1999). Exposure to fungicides in fruit growing: re-entry time as a predictor for dermal exposure. *American Industrial Hygiene Association Journal*.

[12] Canning KM, McQuillan PB, Jablonski W (1998). Laboratory simulation of splashes and spills of organophosphate insecticides on chemically protective gloves used in agriculture. *Annals of Agricultural and Environmental Medicine*.

[13] Wolfe MF, Seiber JN (1993). Environmental activation of pesticides. *Occupational Medicine*.

[14] Clarke EEK, Levy LS, Spurgeon A, Calvert IA (1997). The problems associated with pesticide use by irrigation workers in Ghana. *Occupational Medicine*.

[15] Vikalpani Women's Federation (2003). Self –monitoring of sign and symptoms of pesticide poisoning by farmers occupationally exposed to pesticides. *Study Report*.

[16] Soares W, Almeda R, Sueli M (2003). Rural work and risk factors associated with pesticide use in Minas, Gerais, Brazil. *Cad Saude Publica*.

[17] Kishi M, Hirschhorn N, Djajadisastra M, Satterlee LN, Strowman S, Dilts R (1995). Relationship of pesticide spraying to signs and symptoms in Indonesian farmers. *Scandinavian Journal of Work, Environment& Health*.

[18] Rao P, Arcury T, Quandt S, Doran A (2004). North Carolina growers' and extension agents' perceptions of Latino farmworker's pesticide exposure. *Human Organization*.

[19] WHO Working Group (1989). Cypermethrin. *Environmental Health Criteria*.

[20] Lifeng T, Shoulin W, Junmin J (2006). Effects of fenvalerate exposure on semen quality among occupational workers. *Contraception*.

[21] Colosio C, Barcellini W, Maroni M (1996). Immunomodulatory effects of occupational exposure to mancozeb. *Archives of Environmental Health*.

[22] Nordby K-C, Andersen A, Irgens LM, Kristensen P (2005). Indicators of mancozeb exposure in relation to thyroid cancer and neural tube defects in farmers' families. *Scandinavian Journal of Work, Environment & Health*.

[23] Sanderson WT, Ringenburg V, Biagini R (1995). Exposure of commercial pesticide applicators to the herbicide alachlor. *American Industrial Hygiene Association Journal*.

[24] Hines CJ, Deddens JA, Coble J, Alavanja MCR (2007). Fungicide application practices and personal protective equipment use among orchard farmers in the agricultural health study. *Journal of Agricultural Safety and Health*.

[25] Cantor A, Young-Holt B (2002). Pesticide-related symptoms among farm workers in rural Honduras. *International Journal of Occupational and Environmental Health*.

[26] MacFarlane E, Chapman A, Benke G, Meaklim J, Sim M, McNeil J (2008). Training and other predictors of personal protective equipment use in Australian grain farmers using pesticides. *Occupational & Environmental Medicine*.

[27] Nicol AM, Kennedy SM (2008). Assessment of pesticide exposure control practices among men and women on fruit-growing farms in British Columbia. *Journal of Occupational and Environmental Hygiene*.

[28] Innes DF, Fuller BH, Berger GMB (1990). Low serum cholinesterase levels in rural workers exposed to organophosphate pesticide sprays. *South African Medical Journal*.

[29] Kackar R, Srivastava MK, Raizada RB (1999). Assessment of toxicological effects of mancozeb in male rats after chronic exposure. *Indian Journal of Experimental Biology*.

[30] Senthilselvan A, McDuffie HH, Dosman JA (1992). Association of asthma with use of pesticides: results of a cross-sectional survey of farmers. *American Review of Respiratory Disease*.

[31] Karlsson SI (2004). Agricultural pesticides in developing countries. *Environment*.

[32] Lee BW, London L, Paulauskis J, Myers J, Christiani DC (2003). Association between human paraoxonase gene polymorphism and chronic symptoms in pesticide-exposed workers. *Journal of Occupational and Environmental Medicine*.

